# Atopische Dermatitis – Identifikation von Bedürfnissen der deutschen Bevölkerung mittels Internetsuchanfragen

**DOI:** 10.1007/s00105-022-04974-x

**Published:** 2022-03-16

**Authors:** Linda Tizek, Maximilian C. Schielein, Lucas Tizek, Alexander Zink

**Affiliations:** Fakultät für Medizin, Klinik und Poliklinik für Dermatologie und Allergologie, Technische Universität München, Biedersteiner Str. 29, 80802 München, Deutschland

**Keywords:** Google, Infodemiologie, Neurodermitis, Gesundheitsinformation, Lokalisation, Zeitlicher Trend, Google, Infodemiology, Atopic eczema, Health information, Location, Time trend

## Abstract

**Hintergrund:**

Das Internet ist eine der wichtigsten Informationsquellen für gesundheitliche Themen für die Allgemeinbevölkerung. Deshalb kann die Analyse von Internetsuchmaschinen dabei helfen, die gesellschaftlichen Interessen und Bedürfnisse bezüglich Erkrankungen zu erfassen.

**Ziel:**

Ziel dieser Studie war es, die Suchanfragen zu atopischer Dermatitis (AD) in Bezug auf Häufigkeit, Interessenschwerpunkt und zeitliches Auftreten in allen deutschen Bundesländern zu untersuchen, um mögliche regionale Unterschiede zu identifizieren.

**Material und Methoden:**

Mithilfe des Google Ads Keyword Planner wurden AD relevante Keywords inklusive deren monatlichen Suchvolumens zwischen Januar 2017 und Dezember 2020 identifiziert, die in Interessenschwerpunkte unterteilt wurden.

**Ergebnisse:**

Es wurden 1419 Keywords gefunden, die ein Suchvolumen von 14.817.610 Anfragen hatten. Das größte Suchvolumen hatte die Kategorie *Allgemein *(*n* = 5.970.840), jedoch wurden der Kategorie *Lokalisation* die meisten Keywords zugeordnet (*n* = 348). Rund 60 % der Keywords zu Lokalisation bezogen sich auf AD im Gesicht. Von allen Bundesländern hatten Bremen und Hamburg das größte Suchvolumen pro 100.000 Einwohner. Mit über 70 % war ein enormer Anstieg im Suchvolumen zu beobachten, der v. a. 2020 sichtbar wurde.

**Diskussion:**

Durch diese Internetsuchmaschinenanalyse konnte verdeutlicht werden, welche AD-relevanten Aspekte von besonderer Bedeutung für die Bevölkerung waren, was dabei helfen kann, Informationskampagnen zielgerichtet anzupassen. Zudem unterstreicht die Studie die immer größer werdende Relevanz des Internets als Informationsquelle für gesundheitliche Themen.

**Zusatzmaterial online:**

Die Online-Version dieses Beitrags (10.1007/s00105-022-04974-x) enthält zusätzliche Tabellen. Beitrag und Zusatzmaterial stehen Ihnen im elektronischen Volltextarchiv auf https://www.springermedizin.de/der-hautarzt zur Verfügung. Sie finden das Zusatzmaterial am Beitragsende unter „Supplementary Information“.

## Abstract (en)

**Background:**

The internet is one of the most important sources for health-related information for the general population. Therefore, the analysis of internet search engines can help to capture the social interests and needs regarding diseases.

**Objectives:**

The aim of this study was to investigate the search queries related to atopic dermatitis (AD) regarding frequency, focus of interest and temporal occurrence in the German federal states in order to identify possible regional differences.

**Material and methods:**

Using the Google Ads Keyword Planner, AD relevant keywords including the monthly search volume between January 2017 and December 2020 were identified, which were then assigned to different categories.

**Results:**

Overall, 1419 keywords were found that had a search volume of 14,817,610 queries. The category ‘general’ had the highest search volume (*n* = 5,970,840), but most keywords were assigned to the category ‘location’ (*n* = 348). About 60% of the keywords assigned to ‘location’ were related to AD on the face. On a national level, Bremen and Hamburg had the highest search volume per 100,000 inhabitants. With more than 70%, an enormous increase in search volume could be observed, which was especially high in 2020.

**Discussion:**

With this internet search analysis, it was possible to clarify which aspects of AD were of particular importance for the German population, which can help to adapt information campaigns to the target population. In addition, the study underlines the increasing relevance of the internet as a source of information regarding health-related topics.

Atopische Dermatitis (AD) ist eine häufige chronische Hauterkrankung, die eine lebenslange Belastung der Lebensqualität darstellen kann. Trotz hocheffektiver Behandlungsmöglichkeiten gibt es noch einen erheblichen Anteil betroffener Personen, die keine adäquate medizinische Behandlung erhalten. Deshalb ist es wichtig, auch unkonventionelle Methoden wie Online-Suchmaschinenanalysen zu nutzen, um einen holistischen Überblick über die Interessen und Bedürfnisse der Bevölkerung zu bekommen. Dabei ermöglichen Suchmaschinenanalysen, sowohl regionale als auch zeitliche Trends zu identifizieren.

## Hintergrund

Die AD ist eine chronische Hauterkrankung, deren Prävalenz in den letzten Jahrzehnten zugenommen hat, wobei Kinder häufiger betroffen sind als Erwachsene [17, 23]. In Deutschland beträgt die Prävalenz bei Erwachsenen zwischen 1,3 und 3,7 % [10, 21]. Bei AD, die durch starken Juckreiz und wiederkehrende ekzematöse Läsionen charakterisiert ist, kann die Lebensqualität von Betroffenen enorm eingeschränkt sein [7, 23]. Die psychosoziale Belastung kann sich nicht nur in Depressionen oder suizidalen Gedanken äußern, sondern auch in einem reduzierten Glücksempfinden [6, 7, 14, 23].

Die Krankheit stellt zudem eine finanzielle Belastung dar. Die in Europa durchschnittlichen selbst getragenen Kosten für die Gesundheitsversorgung betragen rund 927,12 € pro Jahr pro betroffener Person, wobei die erheblichen Aufwendungen für Produkte des täglichen Bedarfs, wie z. B. Pflegecremes, nicht inbegriffen sind [28]. Diese hohen Ausgaben sind unter anderem ein Resultat der Nichtinanspruchnahme medizinischer Leistungen, wodurch Betroffene keine adäquate Therapie erhalten können [13], obwohl durch die Zulassung von modernen Therapien die Behandlung von Menschen mit mittelschwerer bis schwerer AD signifikant verbessert werden konnte [6, 24].

Um sich über gesundheitliche Themen unabhängig vom Arztkontakt zu informieren, ist das Internet eine der wichtigsten und am meisten genutzten Quellen [1]. Nicht nur Betroffene, sondern auch deren Angehörige suchen online nach Informationen, um sich beispielsweise über Therapieansätze zu informieren [6]. In Deutschland nutzten 2020 ca. 94 % der über 13-jährigen Bevölkerung das Internet, und mit ca. 90 % ist Google die am häufigste verwendete Suchmaschine [2, 5]. Durch diesen hohen Marktanteil eignen sich die Daten dazu, das Interesse der Bevölkerung zu verschiedenen Aspekten einer Erkrankung zu untersuchen. Frühere Studien konnten z. B. Korrelationen zwischen dem Suchvolumen von Hautkrebs mit Registerdaten zeigen oder nationale und internationale Interessenunterschiede bezüglich Juckreiz identifizieren [9, 20, 27].

Ziel dieser Studie war es, die Suchanfragen zu AD in Bezug auf Häufigkeit, Interessenschwerpunkt und zeitliches Auftreten in allen deutschen Bundesländern zu untersuchen, um mögliche regionale Unterschiede zu identifizieren.

## Methodik

### Studiendesign

Es wurde eine retrospektive longitudinale Studie durchgeführt, die mithilfe des Google Ads Keyword Planner das AD-Suchvolumen zwischen Januar 2017 und Dezember 2020 untersucht. Google Ads wurde ursprünglich implementiert, um Marketingkampagnen zu optimieren, jedoch wurde das Tool immer häufiger auch für wissenschaftliche Fragestellungen genutzt [19, 27]. Die für die jeweilige Fragestellung ausgewählten Keywords werden in das Tool eingepflegt, welches dann eine Liste mit assoziierten und relevanten Keywords (einzelne Wörter oder Phrasen) inklusive deren Suchvolumen liefert. Dabei entspricht das Suchvolumen den Suchanfragen, die pro Monat bei Google generiert werden. In dieser Studie wurden die Begriffe „Neurodermitis“ und „atopische Dermatitis“ dazu verwendet, um das dazugehörige Suchvolumen in allen Bundesländern zu untersuchen. Die Region- und Spracheinstellungen wurden so festgelegt, dass nur Nutzer berücksichtigt wurden, die in den jeweiligen Regionen lebten/sich auf hielten, die die Produkte auf Deutsch verwendeten und die eine deutsche IP-Adresse nutzten. Da für die Studie keine personenbezogenen Daten, sondern öffentlich frei zugängliche Daten verwendet wurden, waren weder ein Ethikvotum noch eine Einwilligungserklärung notwendig.

### Klassifizierung

Alle identifizierten Keywords wurden zunächst qualitativ untersucht und durch ein induktives Vorgehen wurden 10 verschiedene Interessenschwerpunkte identifiziert: (1) *Allgemein* (z. B. „Neurodermitis“), (2) *Differenzialdiagnose* (z. B. „Ist Neurodermitis Schuppenflechte?“), (3) *Einflussfaktor* (z. B. „Atopisches Ekzem Schwangerschaft“), (4) *Information* (z. B. „Hilfe bei Neurodermitis“), (5) *Lebensabschnitt* (z. B. „Atopisches Ekzem Baby“), (6) *Lokalisation* (z. B. „Neurodermitis Gesicht“), (7) *Pflege* (z. B. „Handcreme bei Neurodermitis“), (8) *Symptom* (z. B. „Neurodermitis Juckreiz“), (9) *Therapie* (z. B. „Neurodermitis Behandlung“) und (10) *Verträglichkeit* (z. B. „Neurodermitis Kosmetik“). Die Kategorie *Therapie *wurde zudem in weitere Unterkategorien wie beispielsweise: (a) *Allgemein* (z. B. „Atopische Dermatitis Medikamente“), (b) *Biologika* (z. B. „Biologicals Neurodermitis“), (c) *Cortison* (z. B. „Cortisonsalbe bei Neurodermitis“), (d) *Hausmittel* (z. B. „Hausmittel Neurodermitis Gesicht“), (e) *Lichttherapie* (z. B. „Phototherapie Neurodermitis“), (f) *Marken/Produkte*, (g) *Naturheilkunde* (z. B. „Neurodermitis Naturheilmittel“) und (h) *neue Therapien* (z. B. „Neurodermitis neue Behandlung“) gegliedert. Suchbegriffe wurden mehreren Kategorien zugeordnet, sofern sie mehrere Kriterien erfüllten.

### Statistik

Das Suchvolumen wurde pro 100.000 Einwohner berechnet und deskriptiv ausgewertet [18]. Um zu untersuchen, ob es zeitliche Trends (zwischen den Jahren und zwischen den Jahreszeiten) gab und ob das Suchvolumen in den Bundesländern unterschiedlich war, wurden einfaktorielle Varianzanalysen (ANOVA) mit einem Bonferroni-post-hoc-Test verwendet. Bei Verletzungen der Annahmen wurde die Welch-ANOVA mit einem Games-Howell-post-hoc-Test berechnet. Ausgenommen der Stadtstaaten wurde der Zusammenhang zwischen der Anzahl an Suchanfragen und verschiedenen Variablen (z. B. soziodemografische Merkmale, Bevölkerungsdichte, Ärzte je Einwohner) mithilfe der Korrelation nach Pearson untersucht [2, 4]. Die Stadtstaaten wurden in der Analyse nicht berücksichtigt, da sowohl die urbane als auch die ländliche Bevölkerung betrachtet werden sollten. Für die räumliche Darstellung wurden die digitalen Geodaten des Bundesamtes für Kartographie und Geodäsie mithilfe eines freien Open-Source-Geographischen-Informationssystems (QGIS Version 2.14.22, QGIS Entwicklungsteam, 2016, Minden, Deutschland) dargestellt [3]. Die statistische Auswertung wurde mit SPSS 26 (IBM Corporation, Armonk, NY, USA) durchgeführt.

## Ergebnisse

**Figure 1:**
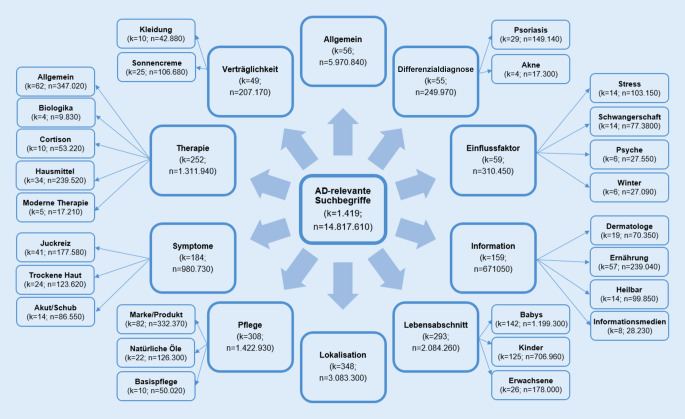

Insgesamt wurden 1419 AD-relevante Keywords identifiziert, die von Januar 2017 bis Dezember 2020 14.817.610 Suchanfragen erzielten (Abb. 1). Das am häufigste gesuchte Keyword war „Neurodermitis“ (*n* = 5.226.810, 36,3 %), gefolgt von „Neurodermitis Baby“ (*n* = 411.470, 2,8 %) und „atopisches Ekzem“ (*n* = 317.830, 2,1 %).

### Kategorisierung

Die Abb. 1 zeigt die Anzahl an Suchbegriffen und deren Suchvolumen in den einzelnen Kategorien. Die Kategorie *Allgemein *hatte mit 5.970.840 Suchanfragen das größte Suchvolumen, obwohl diese Kategorie nur 56 Keywords und somit 3,9 % aller identifizierten Keywords enthielt. Fast gleich viele Keywords wurden der Kategorie *Einflussfaktor* zugeordnet (*n* = 59, 4,2 %), von denen sich jeweils 14 Keywords (23,7 %) auf Stress und Schwangerschaft sowie 6 Keywords (10,2 %) auf die Psyche bezogen. In der Kategorie *Information* (159 Keywords, 11,2 %) bestand das größte Interesse an Ernährung (*n* = 57, 35,8 %). Zudem zeigte sich, dass es in der Kategorie *Lebensabschnitt* (293 Keywords, 20,6 %) viel mehr Keywords zu AD bei Babys (*n* = 142, 48,5 %) als bei Erwachsenen (*n* = 26, 8,8 %) gab. In der Kategorie *Symptom* (180 Keywords, 13,0 %) beschäftigten sich die meisten Keywords mit Juckreiz (*n* = 41, 22,8 %) und trockener Haut (*n* = 24, 13,3 %).

**Figure 2:**
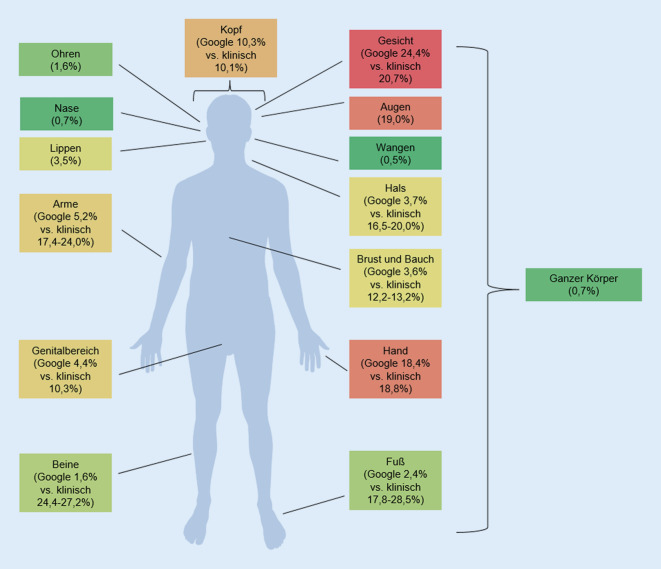

Die meisten Keywords wurden der Kategorie *Lokalisation* zugeordnet (*n* = 348, 24,5 %). Das höchste Suchvolumen wurde für AD im Gesicht (*n* = 716.160, 24,4 %), an den Augen (*n* = 560.370, 19,0 %) und an den Händen (*n* = 540.780, 18,4 %) verzeichnet. Insgesamt machten Lokalisationen im Gesicht 60,0 % der Anfragen in dieser Kategorie aus. Außerdem wurde etwa gleich häufig nach AD an den Armen (*n* = 185.080, 5,2 %) und im Genitalbereich (*n* = 169.050, 4,4 %) gesucht (Abb. 2).

Die Kategorie *Pflege* enthielt 308 Suchbegriffe (21,7 %), wovon sich die meisten Keywords auf bestimmte Marken (*n* = 82, 26,6 %) bezogen und einige auf beispielsweise Öle (*n* = 22, 7,1 %). Im Vergleich dazu wurden rund 50 Keywords weniger der Kategorie *Therapie* zugeordnet (*n* = 252, 17,8 %). Während sich in dieser Kategorie nur 4 Suchbegriffe (1,6 %) auf Biologika und nur 5 (2,0 %) auf neue Therapien bezogen, enthielten 34 Keywords (13,5 %) das Wort Hausmittel (Abb. 1).

### Vergleich zwischen den Bundesländern

**Figure 3:**
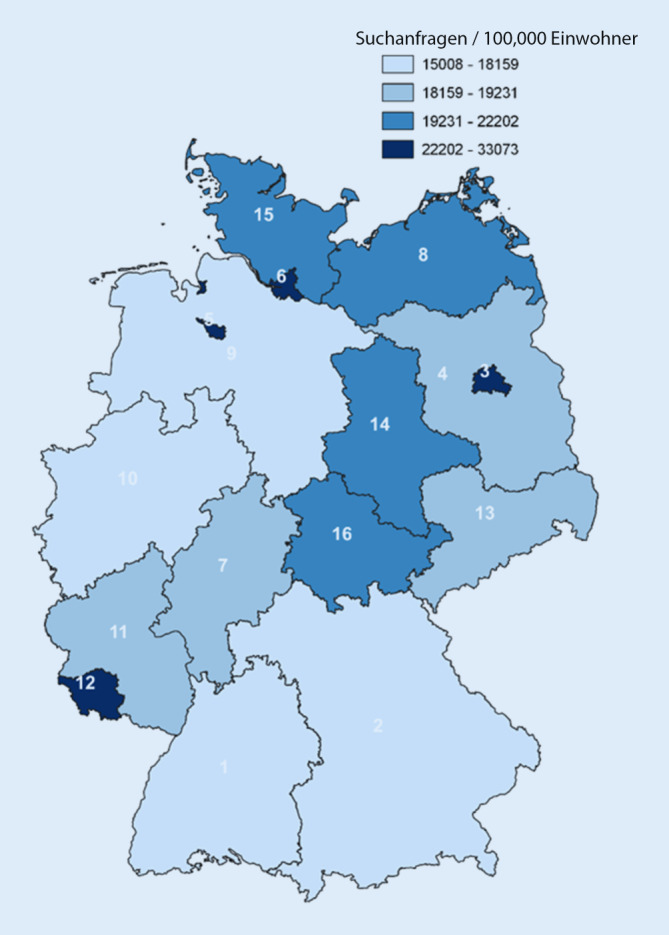

Das höchste Suchvolumen pro 100.000 Einwohner hatten Hamburg (*n* = 33.072,6) und Bremen (*n* = 31.343,6), welches mehr als doppelt so hoch war wie das von Bayern (*n* = 15.007,8) und Nordrhein-Westfalen (NRW, *n* = 15.753,9, Tab. 1, Abb. 3). Die post-hoc Tests zeigten, dass es in jeder Kategorie bis auf *Allgemein* (*p* = 1,00) signifikante Unterschiede im relativen Suchvolumen zwischen den Bundesländern gab. Zum Beispiel unterschied sich in der Kategorie *Differenzialdiagnose* das Suchvolumen pro 100.000 Einwohner in Rheinland-Pfalz (*n* = 366,1) signifikant von dem in Baden-Württemberg (*n* = 218,7; *p* < 0,001), Bayern (*n* = 203,0; *p* < 0,001), Bremen (*n* = 856,1; *p* = 0,001), NRW (*n* = 189,9; *p* < 0,001) und dem Saarland (*n* = 701,1; *p* = 0,031, Tab. 1). Die Korrelationsanalyse ergab, dass die Anzahl an Suchanfragen signifikant positiv korrelierte mit dem Durchschnittsalter (r = 0,560, *p* = 0,047) und der Anzahl an Ärzten je Einwohner (r = 0,589, *p* = 0,034, Online-Tab. 1).

**Table 1:** 

Bundesland	Anfragen pro 100.000	Allgemein	Differenzialdiagnose	Einflussfaktor	Information	Lebensabschnitt	Lokalisation	Pflege	Symptom	Therapie	Verträglichkeit
Baden-Württemberg	15.681,9	7107,7 (45,3 %)	218,7 (1,4 %)	275,8 (1,8 %)	610,3 (3,9 %)	2133,2 (13,6 %)	3128,4 (19,9 %)	1252,2 (8,0 %)	813,8 (5,2 %)	1235,1 (7,9 %)	181,4 (1,2 %)
Bayern	15.007,8	6772,9 (45,1 %)	203,0 (1,4 %)	267,6 (1,8 %)	554,3 (3,7 %)	2160,2 (14,4 %)	2971,8 (19,8 %)	1183,6 (7,9 %)	765,3 (5,1 %)	1143,6 (7,6 %)	182,6 (1,2 %)
Berlin	23.651,7	8994,5 (38,0 %)	471,0 (2,0 %)	544,3 (2,3 %)	1226,5 (5,2 %)	3329,0 (14,1 %)	4745,7 (20,1 %)	2587,9 (10,9 %)	1574,0 (6,7 %)	2281,0 (9,6 %)	367,9 (1,6 %)
Brandenburg	19.024,2	5643,5 (29,7 %)	413,6 (2,2 %)	511,1 (2,7 %)	1149,9 (6,0 %)	3259,7 (17,1 %)	4046,8 (21,3 %)	2463,5 (12,9 %)	1484,5 (7,8 %)	2126,9 (11,2 %)	364,8 (1,9 %)
Bremen	31.343,6	7656,4 (24,4 %)	856,1 (2,7 %)	1058,7 (3,4 %)	2105,7 (6,7 %)	4088,1 (13,0 %)	8195,3 (26,1 %)	4356,8 (13,9 %)	2704,8 (8,6 %)	3699,0 (11,8 %)	543,3 (1,7 %)
Hamburg	33.072,6	10.854,4 (32,8 %)	745,5 (2,3 %)	900,9 (2,7 %)	1955,6 (5,9 %)	4467,8 (13,5 %)	7523,6 (22,7 %)	3863,0 (11,7 %)	2470,5 (7,5 %)	3361,7 (10,2 %)	558,2 (1,7 %)
Hessen	18.730,8	7679,5 (41,0 %)	316,3 (1,7 %)	375,8 (2,0 %)	863,9 (4,6 %)	2539,4 (13,6 %)	3895,8 (20,8 %)	1796,0 (9,6 %)	1144,7 (6,1 %)	1642,8 (8,8 %)	251,7 (1,3 %)
Mecklenburg-Vorpommern	21.718,9	6203,4 (28,6 %)	486,3 (2,2 %)	631,2 (2,9 %)	1341,4 (6,2 %)	3596,4 (16,6 %)	4702,1 (21,6 %)	2909,8 (13,4 %)	1777,4 (8,2 %)	2429,7 (11,2 %)	430,3 (2,0 %)
Niedersachsen	16.682,6	6924,6 (41,5 %)	269,1 (1,6 %)	325,7 (2,0 %)	734,8 (4,4 %)	2256,6 (13,5 %)	3491,7 (20,9 %)	1530,9 (9,2 %)	1003,5 (6,0 %)	1450,8 (8,7 %)	225,2 (1,3 %)
Nordrhein-Westfalen	15.753,9	7609,5 (48,3 %)	189,9 (1,2 %)	260,6 (1,7 %)	479,8 (3,0 %)	1966,4 (12,5 %)	3234,5 (20,5 %)	1100,4 (7,0 %)	738,3 (4,7 %)	1105,6 (7,0 %)	161,9 (1,0 %)
Rheinland-Pfalz	18.694,4	6835,6 (36,6 %)	366,1 (2,0 %)	424,8 (2,3 %)	1006,8 (5,4 %)	2698,6 (14,4 %)	3999,0 (21,4 %)	2026,9 (10,8 %)	1281,6 (6,9 %)	1805,6 (9,7 %)	269,7 (1,4 %)
Saarland	28.019,3	7308,0 (26,1 %)	701,1 (2,5 %)	859,2 (3,1 %)	1708,2 (6,1 %)	3986,8 (14,2 %)	7103,3 (25,4 %)	3826,7 (13,7 %)	2409,3 (8,6 %)	3217,8 (11,5 %)	442,8 (1,6 %)
Sachsen	18.650,8	6553,5 (35,1 %)	359,3 (1,9 %)	432,5 (2,3 %)	1024,8 (5,5 %)	2999,8 (16,1 %)	3673,9 (19,7 %)	2113,7 (11,3 %)	1337,4 (7,2 %)	1938,6 (10,4 %)	350,4 (1,9 %)
Sachsen-Anhalt	19.437,8	5706,2 (29,4 %)	432,8 (2,2 %)	502,5 (2,6 %)	1140,8 (5,9 %)	3233,3 (16,6 %)	4147,2 (21,3 %)	2593,6 (13,3 %)	1581,3 (8,1 %)	2239,2 (11,5 %)	377,7 (1,9 %)
Schleswig-Holstein	20.141,2	6771,3 (33,6 %)	452,1 (2,2 %)	509,0 (2,5 %)	1175,3 (5,8 %)	2848,5 (14,1 %)	4418,0 (21,9 %)	2383,6 (11,8 %)	1534,8 (7,6 %)	2048,6 (10,2 %)	334,7 (1,7 %)
Thüringen	20.364,3	5878,6 (28,9 %)	434,1 (2,1 %)	568,7 (2,8 %)	1235,3 (6,1 %)	3411,6 (16,8 %)	4317,4 (21,2 %)	2716,4 (13,3 %)	1661,5 (8,2 %)	2348,3 (11,5 %)	415,4 (2,0 %)
Durchschnitt	17.816,9	7179,4 (40,3 %)	300,6 (1,7 %)	373,3 (2,1 %)	806,9 (4,5 %)	2506,1 (14,1 %)	3707,4 (20,8 %)	1711,0 (9,6 %)	1092,7 (6,1 %)	1577,5 (8,9 %)	249,1 (1,4 %)

Besonders in den bevölkerungsreichen Bundesländern wie beispielsweise Bayern und NRW wurde mit mehr als 40 % des Suchvolumens ein hoher Anteil an allgemeinen Anfragen verzeichnet. Im Vergleich dazu lag der Anteil im Saarland bei nur 26,1 %, was dort etwa gleich groß war wie der Anteil der Anfragen zu Lokalisationen (25,4 %, Tab. 1). Eine detaillierte Betrachtung der Kategorie *Therapie* zeigte, dass in jedem Bundesland ca. 25 % allgemeine Therapieanfragen waren. Mit 1,2 % verzeichnete Schleswig-Holstein den höchsten Anteil an Anfragen zu Biologika, wohingegen mit 21,3 % der höchste Anteil an Anfragen zu Hausmitteln in Bremen beobachtet wurde (Online-Tab. 2).

### Zeitlicher Verlauf

**Figure 4:**
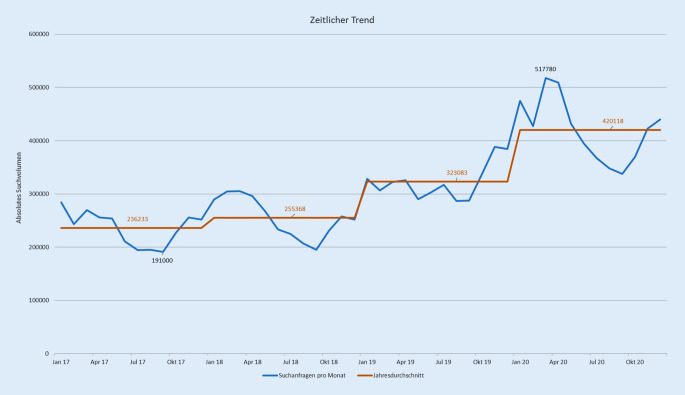

Während des 4‑jährigen Untersuchungszeitraums stieg das Suchvolumen um 78,9 % an, wobei zwischen 2017 und 2018 nur ein sehr leichter Anstieg verzeichnet wurde. In 2019 nahm das Suchvolumen um 24,3 % zu und war signifikant höher als im Vorjahr (*p* = 0,046). In 2020 erhöhte sich das Suchvolumen um weitere 26,2 % auf 5.071.410 Suchanfragen (*p* = 0,001). Generell konnte kein signifikanter Unterschied im Suchvolumen zwischen den Jahreszeiten festgestellt werden (*p* = 0,174, Abb. 4).

## Diskussion

Ziel dieser Studie war es, die Online-Suchanfragen zu AD in Bezug auf Häufigkeit, Interessenschwerpunkt und zeitliches Auftreten in allen deutschen Bundesländern zu untersuchen, um potenzielle regionale Unterschiede zu identifizieren. Mit einem Anstieg von mehr als 70 % gab es einen extremen Zuwachs an Suchanfragen, v. a. in 2020. Fast die Hälfte des Suchvolumens machten allgemeine Anfragen aus, obwohl die meisten Keywords der Kategorie *Lokalisation* zugeordnet wurden. Das Suchvolumen pro 100.000 Einwohner war signifikant höher in Bundesländern mit weniger Einwohnern wie Bremen und Hamburg als in Baden-Württemberg, Bayern und NRW.

Insgesamt wurden fast 15 Mio. Suchanfragen zu AD registriert. Somit wurden mehr Anfragen als zu Juckreiz (rund 14 Mio.) gefunden, jedoch weniger als zu Hautkrebs (knapp 20 Mio.) [15, 26]. Wird jedoch berücksichtigt, dass Juckreiz nicht nur ein häufiges Symptom von AD, sondern auch von Psoriasis und Skabies ist [26] und dass die Prävalenz von nichtmelanozytärem Hautkrebs sehr viel höher ist als die von AD [20, 21], scheint das Suchvolumen von AD verhältnismäßig sehr groß zu sein. Es wurden zwar unterschiedliche Jahre betrachtet, dennoch lässt sich vermuten, dass Betroffene mit AD ein höheres Verlangen haben, sich online über ihre Erkrankung zu informieren, da die Erkrankung oft chronisch oder chronisch rezidivierend ist. Dieses Bedürfnis scheint durch die COVID-19-Pandemie noch einmal deutlich größer geworden zu sein, was ein Resultat davon sein könnte, dass weniger Menschen dermatologische Leistungen in Anspruch genommen haben [22]. Aufgrund der zunehmenden Anzahl an Suchanfragen während der letzten Jahre kann es umso wichtiger werden, vermehrt auch unkonventionelle Maßnahmen wie Internetsuchdaten zu nutzen, um einen holistischen Überblick über die Interessen und Bedürfnisse einer Bevölkerung zu erhalten. Mit diesen Ergebnissen könnten Informationskampagnen zielgerichtet angepasst werden, was einen positiven Effekt auf das Gesundheitsbewusstsein haben kann und somit entscheidend zur Verbesserung der Gesundheitsversorgung beitragen kann [8, 11].

Ähnlich wie bei anderen Studien war der Großteil des Suchvolumens durch allgemeine Suchen bedingt [12, 15, 20]. Gemessen an der Anzahl an Keywords war in dieser Studie das Interesse an AD bei Babys und Kindern fast 5‑mal so hoch wie bei Erwachsenen, was in etwa die berichtete höhere Prävalenz bei Kindern widerspiegelt [17, 23]. Hingegen konträr zur Literatur war die Verteilung der Suchanfragen zu bestimmten Lokalisationen. Während Suchanfragen zu Füßen und Beinen, die als häufig betroffene Körperstellen berichtet wurden, seltener waren, war der Anteil an Suchanfragen zu Lokalisationen im Gesicht mit 60 % doppelt so hoch wie in der Literatur beschrieben [16]. Dies deutet auf einen höheren Leidensdruck sowie den Wunsch nach weiteren Informationen hin, sobald das Gesicht betroffen ist, und demnach sollte bei der medizinischen Versorgung besonders darauf geachtet werden. Zudem waren in dieser Studie fast 10 % der Anfragen zu Pflege und Therapie. In beiden Kategorien waren die häufigsten Suchanfragen zu bestimmten Marken/Produkten, was dafür spricht, dass Menschen nach zusätzlichen Informationen von AD-Produkten suchen [5]. Auffällig war zudem, dass der Anteil an Suchbegriffen bezüglich Hausmitteln fast 4‑mal so hoch war wie der von neuen Therapieoptionen, was beispielsweise auf die Therapieunzufriedenheit von Menschen in Behandlung oder den Informationswunsch von Menschen ohne Kontakt zum Gesundheitssystem hindeuten könnte [6, 13, 22]. Außerdem scheinen viele Menschen noch nicht ausreichend über die neusten, effektiven Therapiemethoden aufgeklärt zu sein [6], was wiederum hinderlich für eine adäquate Gesundheitsversorgung ist. Dementsprechend sollten Möglichkeiten gefunden werden, Betroffene, die z. B. nach Hausmittel zur Behandlung der AD suchen, mit wissenschaftlichen sowie evidenzbasierten Informationen zu versorgen. Mögliche Ansatzpunkte könnten hierbei häufig genutzte Online-Plattformen oder Suchmaschinen sein.

Im Gegensatz zu früheren Studien konnte keine Korrelation mit der Prävalenz festgestellt werden [2, 12, 20]. Außerdem ließ sich keine Assoziation mit dem Frauenanteil oder dem sozioökonomischen Status feststellen, sondern lediglich mit dem Durchschnittsalter und der Anzahl an Ärzten je Einwohner [4]. Dennoch sind die Ergebnisse dabei hilfreich, die Interessen in den Regionen zu studieren. In den größeren Bundesländern scheinen v. a. Leute über allgemeine Suchanfragen zunächst an Informationen zu gelangen, weshalb es sinnvoll erscheint, dass Leuten möglichst direkt vertrauenswürdige und evidenzbasierte Webseiten angezeigt werden [20]. In weiteren Studien wäre es interessant zu untersuchen, ob innerhalb der Bundesländer ein Unterschied im Nutzungsverhalten zwischen der Land- und Stadtbevölkerung zu beobachten ist.

### Limitationen

Es gibt einige Limitationen. Bei dieser Analyse wurden nur Menschen berücksichtigt, die Google verwenden. Zudem nutzen jüngere Leute das Internet häufiger [1]. Dies kann einer der Gründe sein, warum das Suchvolumen pro 100.000 Einwohner in Stadtstaaten deutlich höher war als in größeren Bundesländern wie Bayern, wo viele ältere Leute in ländlichen Regionen leben. Da Google innerhalb des Bundeslands keine Informationen darüber liefert, wo und von wem (z. B. Alter, Geschlecht) die Anfragen generiert wurden, ist nicht ersichtlich, wie viele Suchanfragen von Betroffenen, Angehörigen, Medizinstudierenden, ärztlichem Personal, Pharmafirmen, Gesundheitsämtern oder sonstigen Personen durchgeführt wurden. Obwohl davon auszugehen ist, dass im Gesundheitswesen tätige Personen ihr Wissen größtenteils über andere Kanäle wie z. B. PubMed beziehen, wollen diese wahrscheinlich dennoch wissen, welche Webseiten über Google zu finden sind, um Betroffene adäquat beraten zu können. Dementsprechend könnte die Heterogenität der Bundesländer die Ergebnisse beeinflusst haben.

### Schlussfolgerung

Trotz der Limitationen ist die Analyse von Suchmaschinendaten eine geeignete Methode, die Interessen und Bedürfnisse einer großen Bevölkerungsgruppe zu untersuchen, die im klinischen Alltag nicht in gleicher Weise sichtbar sind. Die Studie konnte zeigen, dass die Anzahl an Suchanfragen bereits 2017 schon sehr hoch war, aber besonders während der COVID-19-Pandemie enorm angestiegen ist, was zum einen die immer größer werdende Bedeutung des Internets als Informationsquelle verdeutlicht und zum anderen das hohe Informationsbedürfnis von Personen mit AD unterstreicht. Die gewonnenen Erkenntnisse könnten genutzt werden, um Informationskampagnen besser an die Zielgruppe anzupassen, um so die Gesundheitsversorgung zu verbessern. Da besonders viele allgemeine Anfragen beobachtet wurden, die keinen Aufschluss über das weitere Suchverhalten geben, sollte dieses in zukünftigen Studien genauer untersucht werden. Zudem sollte untersucht werden, ob viele Menschen bereits vor dem Arztbesuch das Internet für die Informationsgewinnung nutzen, ob das Suchverhalten durch einen ärztlichen Kontakt beeinflusst wurde und ob die Anzahl an Suchanfragen durch ein größeres Angebot von Neurodermitisschulung in den nächsten Jahren stagniert oder sogar wieder sinkt.

## Fazit für die Praxis

- In den letzten Jahren haben sich Personen häufiger über AD im Internet informiert, wobei die Anzahl an Suchanfragen durch die COVID-19-Pandemie im Jahr 2020 noch einmal deutlich zugenommen hat.
- Abgesehen von allgemeinen Anfragen, gab es viele Keywords zu Lokalisationen, wobei der Anteil an Suchanfragen zu sichtbaren Stellen wie dem Gesicht viel höher war als die klinische Häufigkeit, was den Wunsch nach weiterführenden Informationen verdeutlicht, wenn sichtbare Hautstellen betroffen sind.
- In den letzten paar Jahren wurden Suchmaschinenanalysen als eine neue Methode entdeckt, die Interessen einer Bevölkerung zu gesundheitlichen Themen zu studieren. Sie bieten die Möglichkeit, Informationskampagnen und die Gesundheitsversorgung besser an die Bedürfnisse der Bevölkerung anzupassen.

## Supplementary Information




