# Supplementary material for: Atopic dermatitis—identifying needs in the German population by internet search queries
Source: Hautarzt. 2022 Mar 16;73(6):475–84. [Article in German] doi: 10.1007/s00105-022-04974-x (PMC8925291; doi:10.1007/s00105-022-04974-x)
Supplement: Supplementary file 1 [file 105_2022_4974_MOESM1_ESM.pdf]

Zusätzliches Online-Material

Online-Tab. 1: Ergebnisse der Korrelationsanalyse nach Pearson, um mögliche Zusammenhänge zwischen dem online Suchvolumen und möglichen Einflussfaktoren zu untersuchen.

	RSV	Ausländer-anteil[1]	Durchschnitts-alter	Frauen-anteil	Bevölkerungs-dichte	AD Prävalenz	Internetnutzung	Beschäftigungs-quote	Ärzte je Einwohner	Hausärzte	Ländlichkeit
**Relatives Suchvolumen (RSV)[1]**	1										
**Ausländeranteil[2]**	-,319 (p=,288)	1									
**Durchschnittsalter**	,560* (p=,047)	-,919* (p<0,001)	1								
**Frauenanteil**	,481 (p=,096)	-,199 (p=,514)	,227 (p=,457)	1							
**Bevölkerungsdichte**	-,039 (p=,900)	,698* (p=,008)	-,565* (p=,044)	,285 (p=,344)	1						
**AD Prävalenz**	,404 (p=,171)	-,766* (p=,002)	,873* (p<0,001)	,006 (p=,985)	-,389 (p=,189)	1					
**Internetnutzung**	-,264 (p=,384)	,771* (p=,002)	-,848* (p<0,001)	,127 (p=,679)	,456 (p=,117)	-,894** (p<0,001)	1				
**Beschäftigungsquote**	-,271 (p=,371)	-,508 (p=,077)	,447 (p=,126)	-,553* (p=,050)	-,583* (p=,037)	,558* (p=,047)	-,699* (p=,008)	1			
**Ärzte je Einwohner**	,589* (p=,034)	-,235 (p=,439)	,331 (p=,270)	,338 (p=,258)	-,041 (p=,895)	,189 (p=,536)	-,233 (p=,444)	,077 (p=,803)	1		
**Hausärzte**	,344 (p=,250)	-,551 (p=,051)	,491 (p=,088)	-,014 (p=,963)	-,641* (p=,018)	,257 (p=,396)	-,423 (p=,150)	,412 (p=,162)	,731* (p=,005)	1	
**Ländlichkeit**	,015 (p=,960)	-,766* (p=,002)	,629* (p=,021)	-,157 (p=,609)	-,935* (p<0,001)	,422 (p=,151)	-,532 (p=,061)	,527 (p=,064)	,067 (p=,828)	,657* (p=,015)	1

* Die Korrelation ist auf einem Niveau von 0,05 signifikant. AD = atopische Dermatitis

[1] RSV = relatives Suchvolumen (Suchvolumen pro 100.000 Einwohner).

[2] Anteil der Personen, die keine deutsche Staatsbürgerschaft haben.

Online-Tab. 2: Prozentuale Verteilung der Suchanfragen in ausgewählten Subkategorien der Kategorie Therapie in den Bundesländern.[1]

Bundesland	Allgemein[2]	Biologika	Cortison	Hausmittel	Lichttherapie	Marken/Produkte	Naturheilkunde	Neue Therapien
Baden-Württemberg	28,0%	0,6%	3,7%	17,8%	3,6%	23,5%	6,0%	1,2%
Bayern	27,4%	0,7%	3,8%	18,8%	3,6%	23,1%	6,2%	1,2%
Berlin	24,9%	0,9%	4,2%	16,1%	4,0%	25,7%	7,5%	1,5%
Brandenburg	24,9%	0,7%	4,4%	19,6%	4,1%	23,5%	7,9%	1,2%
Bremen	24,9%	0,9%	4,8%	21,3%	5,0%	22,1%	6,0%	1,7%
Hamburg	25,0%	0,9%	4,5%	15,9%	4,5%	25,9%	7,0%	1,7%
Hessen	26,8%	0,8%	4,1%	16,2%	4,0%	25,2%	6,2%	1,3%
Mecklenburg-Vorpommern	24,2%	0,8%	4,1%	20,5%	4,0%	23,2%	7,2%	1,6%
Niedersachsen	26,5%	0,8%	4,2%	17,8%	3,8%	24,5%	6,0%	1,3%
Nordrhein-Westfalen	29,0%	0,7%	3,8%	19,9%	3,2%	22,7%	4,6%	1,1%
Rheinland-Pfalz	26,0%	0,8%	4,3%	16,1%	3,8%	25,4%	7,1%	1,3%
Saarland	24,6%	0,9%	4,7%	19,2%	5,0%	25,1%	6,8%	1,4%
Sachsen	24,9%	0,6%	3,9%	20,0%	3,9%	22,6%	7,2%	1,4%
Sachsen-Anhalt	24,9%	0,6%	4,0%	19,2%	3,7%	24,1%	7,0%	1,6%
Schleswig-Holstein	25,6%	1,2%	4,5%	17,4%	4,3%	24,6%	7,2%	1,4%
Thüringen	24,1%	0,9%	4,1%	18,6%	4,5%	23,2%	7,9%	1,6%

[1] Die kumulativen Prozentangaben je Bundesland können kleiner als 100% sein, da nicht alle Unterkategorien von Therapie aufgeführt sind.

[2] Beinhaltet Suchanfragen, die keine spezifische Therapie enthielten, wie z.B. „atopische Dermatitis Medikamente“ „Behandlung bei Neurodermitis“ oder „Neurodermitis Behandlung Kinder“.
